# Long-term prognostic factors of clinical success after interventional bronchoscopy in patients with scarring central airway stenosis

**DOI:** 10.1186/s12890-021-01434-5

**Published:** 2021-03-01

**Authors:** Kunyan Sun, Hong Zhang, Wei Zhang, Yuan Cheng, Guangfa Wang

**Affiliations:** Department of Respiratory and Critical Care Medicine, Peking University First Hospital, No. 8 Xishiku Street, Xicheng District, Beijing, 100034 China

**Keywords:** Airway obstruction, Bronchoscopy, Outcome, Subglottic stenosis, Smoking

## Abstract

**Background:**

Scarring central airway stenosis (SCAS) is a potentially life-threatening condition with debilitating symptoms. Interventional bronchoscopy is increasingly used to relieve symptoms in patients with SCAS, but recurrent stenosis is frequently observed. Little data exist on the long-term prognosis of interventional bronchoscopy for SCAS. We aimed to assess the prognostic factors of bronchoscopic interventions in patients with SCAS to optimize treatment.

**Methods:**

This was a retrospective study that enrolled 119 consecutive patients with SCAS from January 2010 to April 2019 at our institution. Long-term clinical success was defined as airway stenosis < 50%, no limitation of physical activity, and a stable condition for > 12 months after the last interventional procedure. We compared patients’ demographics, airway stenosis characteristics, and interventional procedures between the successful and unsuccessful groups, and identified significant predictors of long-term outcome with univariate and multivariate logistic regression.

**Results:**

A total of 119 patients with 577 therapeutic bronchoscopies were included. Seventy-five (63%) patients were considered to have long-term clinical success. Older age, male gender, smoking, elevated C-reactive protein level, subglottic stenosis, stent or T-tube implantation, previous interventional treatment, and multiple procedures per year were potentially associated with unsuccessful long-term outcomes in the univariate analysis. Current smoker status (odds ratio [OR] 5.70, 95% confidence interval [CI] 1.35–24.17, *P* = 0.018), subglottic stenosis (OR 4.35, 95% CI 1.31–14.46, *P* = 0.017), and stent implantation (OR 4.96, 95% CI 1.33–18.48, *P* = 0.017) were associated with decreased odds of long-term success in the multivariate logistic regression analysis. Of note, there was no significant difference in odds of success between former smokers and nonsmokers.

**Conclusions:**

Current smoker status, subglottic stenosis, and stent implantation are independent factors associated with reduced long-term efficacy of interventional bronchoscopy for SCAS. Smoking cessation should be encouraged to improve the outcome of therapeutic bronchoscopy.

**Supplementary Information:**

The online version contains supplementary material available at 10.1186/s12890-021-01434-5.

## Background

Scarring central airway stenosis (SCAS) is a life-threatening condition that is predominantly associated with shrinking and scarring in the trachea, left and right main bronchi, and right middle bronchus, which could cause severe dyspnea [1–3]. Many benign etiologies can result in SCAS, such as post-intubation and post-tracheostomy, infectious inflammation, anastomotic stenosis, airway trauma, and foreign body stimulation [4].

Surgical resection and reconstruction used to be the gold standard therapy [5]. However, the surgery is technically challenging, and many patients are either inoperable or unable to tolerate surgery due to their poor condition. Interventional bronchoscopy modalities, including balloon dilation, electrocautery, laser, argon plasma coagulation, cryotherapy, T-tube, and silicone stent implantation, which are less invasive and can rapidly alleviate shortness of breath, have been increasingly used to manage SCAS [6, 7]. A previous study has reported the short-term efficacy of interventional bronchoscopy as up to 98.67% [8]. Nevertheless, SCAS has a high risk of recurrence which occurs in 40–70% of patients [9]. Thus, patients must receive repeated treatments, which significantly influences their quality of life and increases the financial burden on families, rendering it a challenge in interventional pulmonology. Sratakos et al. and Dalar et al. have reported that the long-term efficacy is lower in complex stenosis featuring long stenosis and cartilage involvement [6, 10]. Identifying which subgroups of patients may benefit from the intervention and obtain long-term successful results is paramount. However, little data regarding the long-term prognosis of interventional bronchoscopy for SCAS are available. Therefore, we retrospectively evaluated the long-term clinical outcome in patients who underwent interventional bronchoscopy for SCAS, aiming to assess the prognostic predictors to optimize treatment.

## Methods

### Patients

We retrospectively reviewed all consecutive patients who underwent interventional bronchoscopy due to SCAS at our institution, a tertiary referral center for interventional bronchoscopy in China, between January 2010 and April 2019. The included patients either had contraindications to surgery or decided to opt for interventional bronchoscopy following a discussion with their surgeons. The inclusion criteria were as follows: (1) diagnosed with SCAS based on bronchoscopy; (2) received interventional bronchoscopic treatment; and (3) followed up for at least 12 months. The study excluded patients with intraluminal malignant tumors, extrinsic compression, dynamic stenosis, active infection, systemic diseases such as relapsing polychondritis and granulomatosis with polyangiitis, and those who were either followed up for less than 12 months or lost to follow-up. The first interventional bronchoscopy treatment was arranged if the patient was symptomatic with more than 50% airway stenosis. Subsequent bronchoscopic therapy was considered for patients with recurrent symptoms, a marked decline in pulmonary ventilatory capacity, or as maintenance treatment determined by the clinicians. Oral informed consent was obtained from all participants and the Institutional Review Board of Peking University First Hospital approved this study (2020209).

### Bronchoscopic strategies for the treatment of SCAS

First, an electronic knife or laser was used to cut the scar tissue radially, and rigid bronchoscope or balloon dilation was then conducted to dilate the stenotic airway. Granulation was debrided using electric snare, holmium–yttrium aluminum garnet (Ho:YAG) laser, or cryotherapy. Three 30 to 60-s cycles of cryoablation were given using a freeze–thaw procedure at the granulation site or basilar portion of the scar. After excluding active infection, patients with recurrent stenosis received an intralesional injection of 1 ml diprospan (betamethasone 2 mg/betamethasone dipropionate 5 mg) to prevent restenosis. Silicone stent implantation was performed with recurrent stenosis, and it would be removed if the airway remained stable for at least 1 year or severe stent-related complications appeared. To prevent severe airway laceration, appropriately sized balloons were selected according to the measurement by CT scan. Whenever possible, we avoided treating the stenosis with active infection. Additionally, electrocautery and other thermo-ablations were avoided to minimize further airway injury and granulation hyperplasia.

### Outcome

The primary outcome was long-term clinical success at the last visit. Long-term clinical success was defined as airway stenosis < 50%, no limitation of physical activity, and the diameter of the airway is stable for more than 12 months after the last interventional procedure [11]. Long-term clinical success for patients who received stent implantation was also defined according to the criteria above.

### Data collection

Clinical information including basic demographics, smoking status, pulmonary comorbidities, symptoms, laboratory examinations at baseline, interventional treatment intervals, the total number of procedures, and non-interventional treatments were extracted from hospital electronic medical records. Data on airway stenosis data, procedure-related data, and complications were extracted from the bronchoscopy report. Structured telephone-based interviews were performed in April 2020. We evaluated the degree of airway stenosis defined by the decrease in cross-sectional area by referring to the Myer–Cotton stenosis grading system [12]. Former smokers and current smokers were distinguished by cigarette cessation before the first interventional bronchoscopy. Carbon dioxide (CO_2_) retention was defined as an arterial CO_2_ pressure > 50 mmHg with an arterial oxygen pressure > 60 mmHg on room air. Multiple modalities (such as electrocautery, laser, dilation, cryotherapy, and diprospan injection) could be performed during a single bronchoscopic therapy. The total number of therapies was defined as the number of interventional bronchoscopies regardless of the use of multiple modalities. The cumulative number of each modality was defined as the sum of the use of respective modality in all bronchoscopies regardless of repeated use during one bronchoscopy. Use of antibiotics was recognized as any systemic antibiotic within 72 h after the procedure. The number of procedures per year was calculated by dividing the total number of procedures by the follow-up duration in years.

### Statistical analyses

Continuous variables were expressed as means ± standard deviations or as medians with quartiles if normality was not presumed. Categorical variables were reported as counts and percentages. Student’s *t* test or Mann–Whitney U tests were performed to test the association between the clinical success of interventional bronchoscopy and continuous variables, whereas the Chi-square test or Fisher’s exact test (if expected value ≤ 5) was performed for categorical variables. Univariate and multivariate logistic regressions were conducted with statistically significant predictors of the clinical outcome. Levels of C-reactive protein (CRP) were normalized by log10 transformation in the regression analysis. The significance level of all analyses was set at a *P* value < 0.05. Statistical analysis was conducted using SPSS software (version 26.0; SPSS Inc., Chicago, IL, USA).

## Results

### Patients and baseline characteristics

**Fig. 1:**
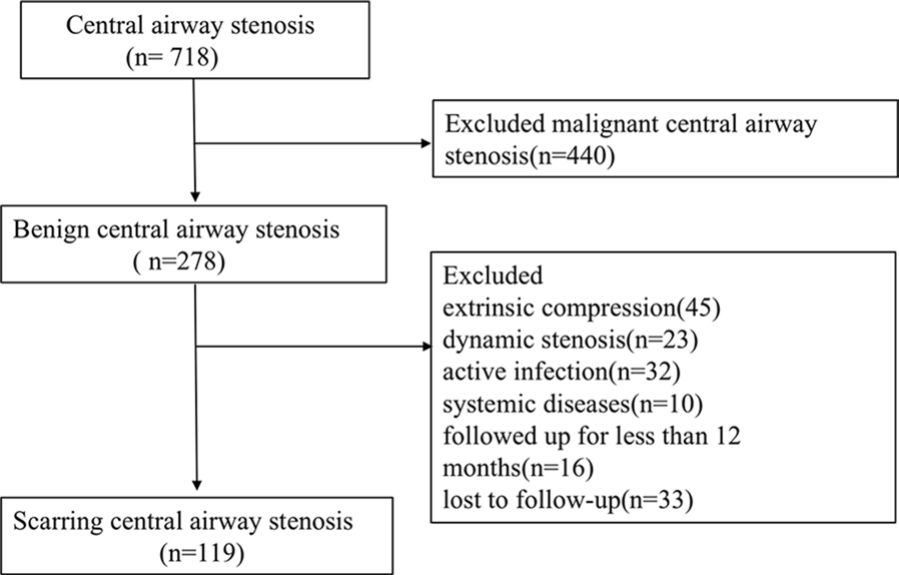
The flow chart of patients’ enrollment

Out of 718 patients with central airway stenosis that underwent interventional bronchoscopic procedures from January 2010 to April 2019, 119 patients (67 males and 52 females) were diagnosed with SCAS and met the criteria for enrollment in the study (Fig. 1). The overall median follow-up time was 53.7 (31.5, 77.0) months, of which 67.9 (42.2, 88.0) and 32.8 (23.5, 53.0) months in the successful and unsuccessful group, respectively. At last follow-up, 75 (63%) patients successfully maintained airway patency for over 12 months without symptoms. The baseline patient characteristics are provided in Table 1.

**Table 1:** Patients’ demographics

Characteristic	All (n = 119)	Successful (n = 75)	Unsuccessful (n = 44)	*P* value
Age (year)	45 (28, 56)	43 (24, 55)	50 (34, 59)	0.034*
Female sex—no. (%)	52 (43.7)	39 (52.0)	13 (29.5)	0.017*
Smoking status				0.035*
Current	26 (21.9)	11 (14.7)	15 (34.1)	
Former	21 (17.6)	13 (17.3)	8 (18.2)	
Nonsmoker	72 (60.5)	51 (68.0)	21 (47.7)	
Underlying lung disease				
COPD	8 (6.7)	6 (8.0)	2 (4.5)	0.709
Asthma	4 (3.4)	2 (2.7)	2 (4.5)	0.626
Lung cancer	9 (7.6)	6 (8.0)	3 (6.8)	1.000
Bronchiectasis	3 (2.5)	3 (4.0)	0	0.295
Symptom				
Cough	51 (42.9)	36 (48.0)	15 (34.1)	0.139
Fever	6 (5.0)	4 (5.3)	2 (4.5)	1.000
Dyspnea	108 (90.8)	68 (90.7)	40 (90.9)	1.000
Chest pain	4 (3.4)	1 (1.3)	3 (6.8)	0.142
WBC (× 10^9^/l)	6.40 (5.10, 7.60)	5.90 (5.24, 7.40)	6.65 (4.68, 8.35)	0.725
Neutrophil percentage (%)	64.8 (56.6, 71.0)	64.5 (56.6, 70.0)	65.4 (56.8, 72.0)	0.928
CRP (mg/l)	2.5 (0.8, 7.8)	2 (0.6, 4.9)	5 (1.1, 10.5)	0.027*
ESR (mm/h)	14 (8, 24)	12 (7, 19)	17.5 (8, 30.8)	0.130
ABG				0.460
Type I respiratory failure	3 (2.8)	3 (4.8)	0 (0)	
Type II respiratory failure	4 (3.7)	2 (3.2)	2 (4.5)	
CO_2_ retention	9 (8.4)	4 (6.3)	5 (11.4)	
FEV1/pred (%)	52.9 ± 20.2	54.9 ± 20.1	48.4 ± 20.1	0.198
PEF/pred (%)	35.7 (18.6, 63.1)	39.0 (19.4, 60.7)	24.4 (15.7, 69.2)	0.494
FVC/pred (%)	79.3 ± 18.8	80.8 ± 19.2	76.0 ± 17.9	0.312
FEV1/FVC (%)	57.8 (38.4, 71.0)	60.1 (38.0, 71.0)	48.1 (39.2, 75.5)	0.666

Data were expressed as count (percentage), mean ± SD or median (interquartile range) as appropriate

*COPD* chronic obstructive pulmonary diseases, *FEV1* forced expiratory volume in one second, *PEF* peak expiratory flow, *FVC* forced vital capacity, *CRP* C-reactive protein, *ESR *erythrocyte sedimentation rate, *WBC* white blood cell, *CO*_*2*_ Carbon dioxide, *ABG* arterial blood gas

*p < 0.05

There were significant differences in terms of age (*P* = 0.034), gender (*P* = 0.017), smoking status (*P* = 0.035), and CRP level (*P* = 0.027) between the successful and unsuccessful groups. The median age of the unsuccessful group was 50 (34,59) years, which was significantly older than that of the successful group (odds ratio [OR] 1.03, 95% confidence interval [CI] 1.00–1.05, *P* = 0.037). Female gender tended to be a protective factor for therapeutic failure compared with male gender (OR 0.39, 95% CI 0.18–0.85, *P* = 0.019). In comparison with nonsmokers, current smokers had higher odds of unsuccessful therapeutic bronchoscopy (OR 3.31, 95% CI 1.31–8.39, *P* = 0.012). Importantly, there was no significant difference in odds of success between former smokers and nonsmokers (OR 1.50, 95% CI 0.54–4.13, *P* = 0.439). Univariate logistic regression analysis showed that elevated CRP increased the risk of therapeutic failure (OR 1.90, 95% CI 1.01–3.57, *P* = 0.046). No significant differences were observed in symptoms, underlying lung disease, white blood cell count, neutrophil percentage, erythrocyte sedimentation rate, pulmonary function test, and arterial blood gas between the two groups.

### Airway stenosis characteristics

The airway stenosis characteristics are summarized in Table 2. The most common etiology of scarring airway stenosis was tracheal intubation or tracheostomy (n = 60, 50.4%), followed by tuberculosis (n = 32, 26.9%), surgical resection (n = 16, 13.4%), unknown reason (n = 7, 5.9%), radiation (n = 2, 1.7%), and trauma (n = 2, 1.7%), without predominance in the two groups. The degree of airway stenosis in the patients clustered between 71 and 99%, while occlusive lesions accounted for 10.9%. There were no significant differences in etiology, stenosis degree, multiple locations of airway stenosis, and airway stenosis length between the successful and unsuccessful groups. Stenosis was most commonly located in the subglottic area (26.9%) and tracheal regions (39.5%), corresponding to the most common etiology, tracheal intubation or tracheostomy. Compared to other stenosis locations, subglottic stenosis was more refractory (OR 6.50, 95% CI 2.67–15.83, *P* < 0.001).

**Table 2:** Airway stenosis and interventional techniques characteristics

Characteristic	All (n = 119)	Successful (n = 75)	Unsuccessful (n = 44)	*P* value
Etiology				0.057
Tracheal intubation or tracheostomy	60 (50.4)	34 (45.4)	26 (59.1)	
Surgery	16 (13.4)	10 (13.3)	6 (13.6)	
Trauma	2 (1.7)	1 (1.3)	1 (2.3)	
TB	32 (26.9)	26 (34.7)	6 (13.6)	
Radiation	2 (1.7)	0	2 (4.6)	
Unknown	7 (5.9)	4 (5.3)	3 (6.8)	
Stenosis degree				0.663
II	33 (27.7)	20 (26.7)	13 (29.6)	
III	73 (61.3)	48 (64.0)	25 (56.8)	
IV	13 (10.9)	7 (9.3)	6 (13.6)	
Location				
Subglottis	32 (26.9)	10 (13.3)	22 (50.0)	< 0.001*
Trachea	47 (39.5)	33 (44.0)	14 (31.8)	0.189
Right main bronchus	16 (13.4)	11 (14.7)	5 (11.4)	0.610
Left main bronchus	29 (24.4)	22 (29.3)	7 (15.9)	0.100
Intermediate bronchus	4 (3.4)	3 (4.0)	1 (2.3)	1.000
Multilocation				0.466
Yes	8 (6.7)	4 (5.3)	4 (9.1)	
No	111 (93.3)	71 (94.7)	40 (90.9)	
Length				0.366
< 1 cm	5 (4.8)	4 (6.1)	1 (2.6)	
1–3 cm	69 (66.3)	46 (69.7)	23 (60.5)	
≥ 3 cm	30 (28.9)	16 (24.2)	14 (36.8)	
Electrocautery	0 (0, 1)	0 (0, 1)	1 (0, 2)	0.089
Laser	0 (0, 0)	0 (0, 0)	0 (0, 1)	0.006*
Mechanical dilation	8 (4, 17)	8 (4, 17)	7 (4, 14)	0.602
Cryotherapy	4 (2, 7)	4 (2, 7)	4 (2, 8)	0.820
Diprospan injection	1 (0, 3)	1 (0, 3)	1 (0, 2)	0.413
Stent				< 0.001*
Yes	23 (19.3)	7 (9.3)	16 (36.4)	
No	96 (80.7)	68 (90.7)	28 (63.6)	
T-tube				0.010*
Yes	7 (5.9)	1 (1.3)	6 (13.6)	
No	112 (94.1)	74 (98.7)	38 (86.4)	
Antibiotics				0.526
Yes	55 (46.2)	33 (44.0)	22 (50.0)	
No	64 (53.8)	42 (56.0)	22 (50.0)	
Time to intervention (days) from diagnosis	22 (7, 72)	22 (8, 63)	28 (7, 92)	0.884
Previous interventional treatment				0.022*
Yes	34 (28.6)	16 (21.3)	18 (40.9)	
No	85 (71.4)	59 (78.7)	26 (59.1)	
Number of procedures per year	1 (0.5, 1.7)	0.8 (0.4, 1.2)	1.5 (0.8, 2.6)	< 0.001*
Interval to second procedure (days)	21 (9, 342)	21 (9, 269)	25 (9, 400)	0.934
Stenosis degree change after first intervention (%)	45 (30, 60)	40 (20, 60)	50 (38, 70)	0.140

Data were expressed as count (percentage) or median (interquartile range) as appropriate

*TB* tuberculosis

*p < 0.05

### Interventional bronchoscopic modalities

The associations of the procedural modalities and long-term outcome were listed in Table 2. The total number of therapeutic interventions performed during follow-up was 4 (2, 7) in the successful group and 5 (2, 7) in the unsuccessful group. Except for laser (*P* = 0.006), there were no differences in the use of electrocautery, mechanical dilation, cryotherapy, diprospan injection, or antibiotics between the two groups. The association with laser disappeared in the univariate analysis (OR 1.68, 95% CI 0.95–2.97, *P* = 0.074). Since there were more patients with tuberculosis in the successful group than in the unsuccessful group (34.7% vs 13.6%), subgroup analysis confined to patients without tuberculosis (n = 87) was performed and revealed less prescription of antibiotics in the successful group (38.8% vs 50.0%). Nevertheless, the difference was not statistically significant (*P* = 0.295). Patients with stent (OR 5.55, 95% CI 2.06–14.96, *P* = 0.001) or T-tube implantation (OR 11.68, 95% CI 1.36–100.59, *P* = 0.025) were more likely to be associated with unsuccessful outcome. The number of procedures per year was significantly higher in the unsuccessful group than that in the successful group, and was associated with higher odds of failure (OR 2.04, 95% CI 1.36–3.06, *P* = 0.001).

Twenty-three (19.3%) patients received 26 stent placements, all of which were silicone stents, including 4 Y-shaped and 22 straight stents. Sixteen stents were removed after 15.4 ± 8.7 months. Seven and 16 patients with stent implantation were in the successful group and unsuccessful group, respectively. All those in the successful group had their stents removed, whereas 6/16 (37.5%) had their stents removed in the unsuccessful group (*P* = 0.007). There was no difference in the duration from stent placement to removal between the two groups (16.4 ± 5.4 m vs 14.6 ± 10.9 months, respectively).

### Variables associated with success of therapeutic bronchoscopy

We assessed all the above factors that were associated with long-term outcome using multivariate logistic regression analysis (Additional file 1: Table S1). Current smoker status (OR 5.70, 95% CI 1.35–24.17, *P* = 0.018), subglottic stenosis (OR 4.35, 95% CI 1.31–14.46, *P* = 0.017), and stent implantation (OR 4.96, 95% CI 1.33–18.48, *P* = 0.017) were associated with lower odds of success after controlling for other variables.

To control the confounding effect of follow-up time, we performed a subgroup analysis including 57 patients from July 2014 to July 2018 (Additional file 1: Table S2). The follow-up time was comparable between the two groups (45.5 vs 39.5 months, respectively). The risk of subglottic stenosis and stent implantation remained independent, while that of current smoking status was significant at the 10% level.

### Complications

Over the 577 interventional bronchoscopies, most of the therapies were performed without significant complications (90.5%). The complications were all manageable, including bleeding requiring adrenaline spraying or electrocoagulation (2.8%), pneumomediastinum or pneumothorax (0.4%), CO_2_ retention (0.2%), fever (0.4%), secretion retention (0.6%), severe bronchial laceration (2.1%), stent migration (1.6%), and stent-related granulation hyperplasia (1.4%). There was no interventional bronchoscopy-related death at our institution.

## Discussion

Interventional bronchoscopy can rapidly improve short-term efficacy by relieving symptoms and airway obstruction [13]. However, few studies have explored the long-term outcomes and prognostic factors of therapeutic bronchoscopy. In this study, 119 patients with SCAS from January 2010 to April 2019 were retrospectively reviewed. We found that 63.0% of the patients maintained long-term stability. Long-term failure of bronchoscopic interventions for SCAS was associated with current smoker status, subglottic stenosis, and stent implantation.

In this study, the female gender was associated with higher odds of clinical success of interventional bronchoscopy. A better therapeutic effect was observed in females. Similarly, in a series of 115 patients with postintubation tracheal stenosis (PITS), Freitas et al. [14] found that simple PITS occurred more frequently in females, whereas complex PITS was more common in males. These findings imply that the therapeutic outcome varies with gender in SCAS.

Another significant finding in the study is that cigarette smoking is related to a higher probability of delayed long-term efficacy. Compared to nonsmokers, current smokers had five times the odds of unsuccessful therapeutic bronchoscopy. Notably, such a difference disappeared between former smokers and nonsmokers. A previous study of patients with laryngotracheal stenosis reported that smoking was associated with tracheostomy dependence and shorter intervals between therapeutic procedures [15]. In addition to benign airway stenosis, Giovacchini et al. [16] found that nonsmokers and former smokers had a higher success rate of therapeutic bronchoscopy than current smokers in malignant conditions. Several studies had proved that smoking could increase airway epithelial inflammation and induce pathologic wound healing [17, 18]. Similarly, it is very likely that smoking could aggravate the injury to the airway mucosa, leading to worse outcomes in SCAS. In accordance with other studies showing worse outcomes in active smokers [19, 20], we confirm cigarette smoking is correlated with poor post-procedural prognosis, and recommend that smoking cessation should be incorporated into future guidelines regarding the management of SCAS.

The most common cause of SCAS in this study was stenosis after tracheal intubation or tracheostomy. A previous study reported that tuberculosis was the most common cause of SCAS in Chinese patients, whereas tracheal intubation or tracheostomy were more common in western countries [21, 22]. We hypothesize that the change in the etiology spectrum is primarily due to the decreased incidence of tuberculosis, which could be attributed to standardized Bacillus Calmette–Guérin vaccination and anti-tuberculosis treatment. Accordingly, the Global Tuberculosis Report 2017 indicated that the global tuberculosis incidence was decreasing every year [23].

Additionally, we found that subglottic stenosis was associated with a significantly lower probability of long-term therapeutic success, in agreement with our clinical experiences. The recurrence rate of subglottic stenosis requiring recurrent endoscopic therapy had been reported to be as high as 40–70% [24]. Hseu et al. conducted a 10-year review of 92 adults with subglottic stenosis, and found that 55% of patients required multiple procedures [25]. Nair et al. [26] reported that the incidence of subglottic decannulation was 68.8% lower than those of glottic and supraglottic stenosis. The subglottic area borders from 5 to 10 mm beneath the vocal folds to the inferior rim of the cricoid cartilage, and the diameter of the cricoid is equivalent to the left main bronchus [27]. Its narrow lumen and special anatomical architecture might make it predisposed to temporized success. Gelbard et al. [28] reported that the iatrogenic etiology of prolonged intubation was one of the most important causes of subglottic stenosis in adults. Therefore, intubation with a proper-sized endotracheal tube, avoiding excessive intra-cuff pressure, and opportune ventilator weaning should be emphasized to minimize injury to the subglottic area.

In this study, for the first time, we demonstrated that CRP might be a candidate predictor of interventional therapeutic outcome. Elevated CRP levels were associated with decreased odds of therapeutic success. CRP is an acute-phase protein and sensitive biomarker of systemic inflammation, primarily secreted by hepatocytes upon stimulation from interleukin (IL)-6 and TNF-α [29, 30]. A previous animal study had confirmed that repeated intubation could result in significant tracheal injury and elevated IL-6 levels both in serum and tracheal tissues [31]. Overexpressed IL-6 upregulates its downstream targets including JAK1, STAT3, RAF1, and ELK1, and induces fibroblastic proliferation and excessive collagen deposition, leading to scar formation [32]. In addition, CRP had been considered as a biomarker of renal and cardiac inflammation and fibrosis [33, 34]. Hence, we speculate that CRP levels correlate positively with the process of airway injury and scar formation, and may serve as a potential serologic marker of SCAS. Yet the underlying association is incompletely understood, which warrants further investigation.

Combined modalities were applied in most of our cases. Thermal therapy has been reported to cause significant granulation tissue growth and cartilage damage compared with mechanical dilation and cryotherapy [35–38]. Thus, we avoided using these modalities where possible to minimize damage to the airway. As scar tissue is resistant to cryotherapy [39], a high frequency electric needle knife or laser was used to cut the scar open radially only when it was too difficult to dilate. Therefore, for non-stent interventional modalities, balloon or rigid bronchoscopy dilation and cryotherapy were the most common techniques we used. In terms of the stent, 23 (19.3%) patients received silicone stent implantation, and the rate of stent implantation was significantly higher in the unsuccessful group. An explanation for this lies in the notion that the patients who were considered for stent implantation in our cohort were considerably sicker and had poor condition or recurrent and intractable stenosis, making the intervention more likely to fail. Moreover, stent implantation may increase the extent of injury and the length of stenosis [38]. Long-term stent implantation is known to be associated with various complications, such as sputum retention, stent migration, and granulation tissue formation [38, 40, 41], which could induce airway restenosis and demand close bronchoscopic surveillance. Intractable stenosis often requires more frequent interventions and multiple procedures to maintain airway patency, and conversely, frequent interventions and multiple procedures can induce secondary airway damage and restenosis, causing therapeutic failure.

Long-term therapeutic success was achieved in 63.0% of the patients in our study, which was identical to previous data reported by Wang et al. [21]. Overall, 90.5% of procedures were conducted without significant complications, and there was no interventional bronchoscopy-related death at our institution. All the above indicate that interventional bronchoscopy is safe and effective for scarring airway stenosis. Our study identified predictors of poor outcome of interventional bronchoscopy, highlighting the crucial role of interventional procedures in selected cases. For those who are unlikely to benefit from the intervention, surgical therapy may be considered in clinical decision-making.

Our study had several limitations. First, it was a retrospective study. Some missing data were inevitable, even if we tried to avoid this eventuality when collecting data. Second, the analysis only included treatment information at our institution. Information of previous treatment(s) at other institutions was not collected due to the lack of detailed records. This might have biased the influence of therapies. Third, the study was conducted at a single institution. A multicenter study is necessary to validate our findings in the future. Finally, the difference in follow-up time between the two groups may have affected long-term success to an extent. We have tried our best to control the potential confounding by including follow-up time as a covariate and conducting subgroup analysis. Further research is needed to assess whether the predictors of long-term therapeutic bronchoscopy success for SCAS in our study hold true in prospective studies.

## Conclusions

In conclusion, interventional bronchoscopy was safe and effective for patients with SCAS. Patients who were current smokers, had subglottic stenosis, and those requiring stent implantation were more likely to experience delayed long-term effectiveness. Smoking cessation should be recommended to optimize the outcomes of therapeutic bronchoscopy.

## Supplementary Information

**Additional file 1.** Variables Associated with Therapeutic Bronchoscopy.

## Data Availability

The datasets used and/or analyzed during the current study are available from the corresponding author on reasonable request.

## Abbreviations

CI: Confidence interval
CO_2_: Carbon dioxide
CRP: C-reactive protein
IL: Interleukin
OR: Odds ratio
PITS: Postintubation tracheal stenosis
SCAS: Scarring central airway stenosis
