# Supplementary material for: Long-term prognostic factors of clinical success after interventional bronchoscopy in patients with scarring central airway stenosis
Source: BMC Pulm Med. 2021 Mar 1;21:73. doi: 10.1186/s12890-021-01434-5 (PMC7923499; doi:10.1186/s12890-021-01434-5)
Supplement: Supplementary file 1 — Additional file 1. Variables Associated with Therapeutic Bronchoscopy. [file 12890_2021_1434_MOESM1_ESM.docx]

**Additional file 1: Variables Associated with Therapeutic Bronchoscopy**

**Additional file 1: Table S1. Variables Associated with Unsuccessful Therapeutic Bronchoscopy: Unadjusted and Adjusted Logistic Regression Analysis (n=119)**

| Characteristic | Unadjusted Odds Ratio | *p* value | Adjusted Odds Ratio^a^ | *p* value |
| --- | --- | --- | --- | --- |
| Age | 1.03 (CI 1.00-1.05) | 0.037^*^ | - | - |
| Female | 0.39 (CI 0.18-0.85) | 0.019^*^ | - | - |
| Smoking status |  |  |  |  |
| Current smoker vs nonsmoker | 3.31 (CI 1.31-8.39) | 0.012^*^ | 5.70 (CI 1.35-24.17) | 0.018^*^ |
| Former smoker vs nonsmoker | 1.50 (CI 0.54-4.13) | 0.439 | 0.42 (CI 0.1-1.75) | 0.232 |
| CRP^#^ | 1.90 (CI 1.01-3.57) | 0.046^*^ | - | - |
| Subglottis | 6.50 (CI 2.67-15.83) | <0.001^*^ | 4.35 (CI 1.31-14.46) | 0.017^*^ |
| Laser | 1.68 (CI 0.95-2.97) | 0.074 | - | - |
| Stent | 5.55 (CI 2.06-14.96) | 0.001^*^ | 4.96 (CI 1.33-18.48) | 0.017^*^ |
| T-tube | 11.68 (CI 1.36-100.59) | 0.025^*^ | - | - |
| Previous interventional treatment | 2.55 (CI 1.13-5.78) | 0.024^*^ | - | - |
| Number of procedures per year | 2.04 (CI 1.36-3.06) | 0.001^*^ | - | - |
| Follow-up time | 0.97 (CI 0.95-0.98) | <0.001^*^ | 0.97 (CI 0.95-1.00) | 0.030^*^ |

### CI=Confidence interval. CRP = C-reactive protein. ^#^Levels of CRP was normalized by log10 transformation. ^a^Adjusted for age, gender, smoking status, log 10 (CRP), subglottis, laser, stent, T-tube, previous interventional treatment, number of procedures per year, follow-up time. **p* < 0.05.

### **Additional file 1: Table S2. Variables Associated with Unsuccessful Therapeutic Bronchoscopy in the subgroup from July 2014 to July 2018 (n=57)**

| Characteristic | Unadjusted Odds Ratio | *p* value | Adjusted Odds Ratio^a^ | *p* value |
| --- | --- | --- | --- | --- |
| Age | 1.04 (CI 1.00-1.08) | 0.046^*^ | - | - |
| Female | 0.28 (CI 0.09-0.88) | 0.029^*^ | - | - |
| Smoking status |  |  |  |  |
| Current smoker vs nonsmoker | 3.83 (CI 0.81-18.09) | 0.090 | ~~-~~ | ~~-~~ |
| Former smoker vs nonsmoker | 1.64 (CI 0.45-6.00) | 0.452 | ~~-~~ | ~~-~~ |
| CRP^#^ | 1.88 (CI 0.88-3.99) | 0.103 | - | - |
| Subglottis | 5.6 (CI 1.62-19.42) | 0.007^*^ | 5.17 (CI 1.36-19.62) | 0.016^*^ |
| Laser | 2.3 (CI 1.01-5.24) | 0.048^*^ | - | - |
| Stent | 5.6 (CI 1.61-19.42) | 0.007^*^ | 5.17 (CI 1.36-19.62) | 0.016^*^ |
| T-tube | 4.57 (CI 0.45-46.95) | 0.201 | ~~-~~ | ^~~-~~^ |
| Previous treatment | 1.95 (CI 0.65-5.81) | 0.233 | ~~-~~ | ^~~-~~^ |
| Number of procedures per year | 2.29 (CI 1.13-4.67) | 0.022^*^ | ~~-~~ | ^~~-~~^ |

**CI=Confidence interval. CRP = C-reactive protein. ^#^Levels of CRP was normalized by log10 transformation. ^a^Adjusted for age, gender, smoking status, subglottis, laser, stent, number of procedures per year. **p* < 0.05**
